# Assessing current and future demand for PET-CT imaging in England: a comparative analysis of 2013 and 2022 Royal College of Radiologists Guidelines

**DOI:** 10.1093/bjro/tzag006

**Published:** 2026-03-04

**Authors:** Nadia A S Smith, Rebecca Nutbrown, Alba Eisner, Raj Jena

**Affiliations:** Nuclear Technologies, TÜV SÜD UK, Warrington, WA3 6AE, United Kingdom; Scientific Computing, Royal Surrey NHS Foundation Trust, Guildford GU2 7XX, United Kingdom; Nuclear Technologies, TÜV SÜD UK, Warrington, WA3 6AE, United Kingdom; Department of Physics, University of Surrey, Guildford GU2 7XH, United Kingdom; Department of Oncology, University of Cambridge, Addenbrooke’s Hospital, Cambridge, CB2 0QQ, United Kingdom; Department of Oncology, University of Cambridge, Addenbrooke’s Hospital, Cambridge, CB2 0QQ, United Kingdom

**Keywords:** PET-CT demand, predictive modeling, healthcare planning, data standardization, cancer, dementia

## Abstract

**Objectives:**

This study aimed to provide a comprehensive analysis of the increasing demand for PET-CT imaging in England, driven by advancements in technology and updated clinical guidelines. By comparing the 2013 and 2022 guidelines issued by the Royal College of Radiologists, the analysis sought to quantify the gap between actual PET-CT provision and modeled requirements, benchmarked against international comparators, to inform strategic planning for equitable access to advanced imaging.

**Methods:**

A detailed modeling approach was developed, mapping guideline-based PET-CT scan requirements to disease incidence and stage for major oncological indications (prostate, lymphoma, neuroendocrine tumors, lung, breast, gynecological cancers) and key non-oncological conditions (dementia, cardiovascular disease). Actual scan volumes were extracted from the NHS Diagnostic Imaging Dataset and cross-referenced with provider data. Unmodeled indications were estimated using proportional scaling. Model outputs were validated against PET-CT utilization rates in Finland, adjusted for UK disease incidence. Future demand projections incorporated population growth, demographic shifts, and evolving disease prevalence.

**Results:**

For 2021, modeled estimates indicated a substantial shortfall in PET-CT provision for several high-impact indications, notably prostate cancer, dementia, neuroendocrine tumors, and breast cancer, where actual scan volumes were 2-7 times lower than modeled need. Modeled demand for England (361 900 scans/year) exceeded actual provision (239 367 scans/year) by over 50%. Comparative analysis with Finland confirmed that England’s PET-CT rates lagged for most indications except lung cancer. Projections suggest continued growth in demand, particularly for dementia and neuroendocrine tumors, with overall PET-CT needs expected to rise sharply by 2040.

**Conclusions:**

England’s current PET-CT capacity falls significantly short of evidence-based requirements, especially for emerging indications in neurology and oncology. Without strategic investment and expansion, this gap will widen, risking delayed diagnosis and suboptimal care. Enhanced data collection and ongoing validation are critical for responsive service planning.

**Advances in knowledge:**

This is the first study to comprehensively model PET-CT demand in England across multiple disease categories using updated clinical guidelines and international benchmarking. It provides a robust, data-driven framework for forecasting imaging needs, supporting national policy and equitable resource allocation.

## Introduction

Nuclear medicine imaging is essential in managing complex conditions and advancing medical research, ultimately improving patient outcomes. Global demand is steadily increasing due to demographic changes, rising disease prevalence, and evolving healthcare needs. In developed countries, the use of nuclear medicine diagnostics is growing at a rate of over 10% annually,^1^ driven by advancements in imaging technology and an expanding range of clinical applications. In particular, Positron Emission Tomography—Computed Tomography (PET-CT) scanning is being increasingly adopted across various medical specialties, including cancer, neurology, and cardiology. In the UK, demand for PET-CT imaging procedures continues to rise, with approximately 280 000 procedures performed annually in England alone.^2^ Despite this growth, research indicates that the UK lags behind other countries with similar Human Development Index (HDI) scores^3^ in terms of PET-CT capacity. This gap underscores the need for greater investment and strategic planning to meet the rising healthcare demands and ensure equitable access to these vital, life-saving technologies.

In England, the use of PET-CT services is primarily guided by recommendations issued in 2013 by the Royal College of Radiologists (RCR).^4^ These guidelines provide evidence-based criteria for the appropriate use of PET-CT, focusing on clinical indications across various medical fields. While these guidelines have shaped current practices and service planning, they are now somewhat outdated given advancements in technology, new clinical evidence, and evolving healthcare needs. Recognizing this, the RCR released updated guidelines in 2022,^5^ reflecting the latest evidence and expanding the indications for PET-CT use in diagnostics and treatment planning.^6–9^ Additionally, interim guidance from National Health Service (NHS) England on the use of PET-CT imaging for prostate cancer is expected. This is particularly important, as prostate cancer is one of the more prevalent cancers in the UK,^10^ and expanded use of PET-CT in its management could lead to a substantial increase in demand.

The primary objective of this research is to estimate the increased demand for PET-CT imaging capacity in England in light of the updated 2022 clinical guidelines.^5^ The current and future projections will consider the growing requirements in oncology, cardiology, and neurology, driven by an aging population and higher disease prevalence. The study will be validated by comparing the estimates with data from countries with similar healthcare systems and HDI levels, providing a robust framework for modeling PET-CT demand. To the best of our knowledge this is the first paper modeling the current and future demand in England for several disease categories. An international comparative analysis was published in 2021,^11^ looking at the differences in PET-CT services across seven countries, including the UK, to understand the impact variation may have upon cancer services.

## Methods

### Clinical conditions modeled

#### Oncological indications

Key oncological indications were specifically chosen to be modeled in detail to accurately assess the true demand for PET-CT imaging capacity. This selection was based on 2022 clinical guidelines,^5^ as well as insights from medical experts (this same methodology was used in Lynch et al.^11^) with the aim of identifying cancers that either have a high anticipated requirement for PET-CT imaging or are classified as high-volume cancers with significant incidence rates. The cancers selected for explicit modeling were:

- **Prostate:** Prostate cancer is one of the most common cancers in men, with high incidence rates globally and in the UK. There is an increasing reliance on PET-CT imaging, especially for detecting metastatic disease and guiding treatment decisions, making it a significant contributor to overall imaging demand.^9^^,^^12^^,^^13^
- **Lymphoma:** Both Hodgkin’s and non-Hodgkin’s lymphoma require frequent PET-CT imaging for staging, treatment planning, and monitoring response to therapy. PET-CT scans are essential in determining both the extent and metabolic activity of the disease (often underestimated on conventional CT), making lymphoma a key focus and tracking treatment effectiveness, making lymphoma a key focus for imaging capacity planning.
- **Neuroendocrine tumors (NETs):** NETs are rare, however, their incidence has increased significantly over recent decades, although this growth is now slowing. These cancers are complex and often require advanced imaging techniques, such as PET-CT, to determine the tumor’s location and extent of spread. PET-CT is especially valuable for detecting tumors that may be missed by other imaging methods, highlighting the need for dedicated imaging capacity.
- **Lung:** As one of the leading causes of cancer-related deaths worldwide, lung cancer has a high incidence rate and frequently requires PET-CT imaging for staging and treatment decision-making. PET-CT plays a crucial role in differentiating between operable and inoperable cases, further underscoring the need for significant imaging resources.
- **Breast:** Breast cancer is the most commonly diagnosed cancer in women and often requires PET-CT imaging for advanced stages, particularly for detecting metastatic disease. Given the high volume of breast cancer cases and the increasing use of PET-CT in treatment planning, its inclusion is vital for understanding the true demand on imaging services.^9^
- **Gynecological:** This category includes cancers such as ovarian, cervical, and uterine cancers. PET-CT is frequently used in staging and monitoring disease progression in advanced gynecological cancers. With these cancers being among the more common cancer types, their inclusion helps account for a significant portion of the overall PET-CT demand.

#### Non-oncological indications

In 2005, the Department of Health published a framework for developing PET services in England.^14^ The document highlighted that while PET is well-established in oncology, its use in cardiology and neurology is less developed. Expert guidance suggested that, in the near future, cancer would account for approximately 85%-90% of PET scan utilization, with only a small percentage of scans needed for neurological and cardiac conditions. Current NHS England clinical commissioning policy allows PET-CT scans for non-oncological indications, as outlined in the *Evidence Based Indications for the Use of PET-CT in the UK 2015 guidelines*,^15^ up to a threshold of 10% of total oncology activity.

In 2021/2022 the proportion of PET-CT scanning conducted for non-oncological conditions was approximately 13%. However, responders to a survey on brain PET and SPECT imaging conducted in 2023^16^ commented on the lack of access to PET imaging in their sites and the limited capacity of PET scanners, as scanning slots were reserved for imaging in oncology. A similar predictive model to that developed for specific oncological indications was created for dementia, incorporating the RCR 2022 guidelines^5^ and input from clinical experts. Key considerations included the increasing use of PET-CT imaging to detect early signs of neurodegenerative diseases, such as Alzheimer’s, as recommended in the guidelines. The model also factored in the anticipated rise in PET-CT demand that is expected once dementia therapies receive approval from the National Institute for Health and Care Excellence (NICE), which is expected in the near future. Such therapies would likely require PET-CT scans for patient selection, monitoring treatment effectiveness, and disease progression. This would significantly expand the role of PET-CT in dementia care, leading to a substantial increase in scan volumes, beyond what is currently used for diagnostic purposes.^9^^,^^14^^,^^17^^,^^18^

A literature review for cardiovascular PET-CT did not reveal any anecdotal evidence of a PET-CT capacity shortage.^9^ However, a comparison between data from England and Finland (see “Comparison to Finland” section) showed that England performs less than half the number of PET-CT scans as Finland. We note that cardiac PET-CT services in Finland, particularly in large centers, often use highly integrated protocols combining perfusion, metabolism, and coronary CT angiography, which may not be directly replicable in the UK due to differences in expertise and organizational structures. Nonetheless, Finnish utilization rates were used as a benchmark for mature adoption of PET-based cardiovascular imaging. These data were adjusted for relative disease incidence to estimate potential UK demand, acknowledging that the resulting figures represent an upper-bound estimate of clinically indicated activity rather than immediate deliverable capacity within the current UK service configuration.

#### The rest

The clinical indications that were not explicitly modeled in the previous subsections, including both oncological and non-oncological conditions, account for 39% of the total PET-CT scans (based on current usage data, obtained from NHS England^2^) These include conditions such as melanoma, sarcoma, head and neck cancers, esophageal and pancreatic cancers, as well as other less common neurological and oncological conditions, which represent a significant portion of the overall demand for PET-CT imaging; but due to the diverse range of conditions and lack of strong consensus guidance, they were not examined in the same detailed manner as the primary clinical indications.

To estimate the true demand for PET-CT scans for these unaccounted clinical indications, a proportional scaling approach was used, which compares the ratio of true demand to the actual number of procedures performed for the clinical indications that were explicitly modeled. This ratio reflects the gap between current procedural volumes and the actual need for PET-CT scans based on clinical guidelines and expert recommendations.

By applying this ratio as a scaling factor, the estimated demand for PET-CT scans for the unmodeled indications can be adjusted upwards to more accurately reflect the actual need for these scans. This method allows for a more comprehensive projection of total PET-CT scan requirements, ensuring that those clinical indications not directly modeled are adequately represented in terms of their potential demand on imaging resources.

### Methodology to calculate estimated demand

**Figure 1:**
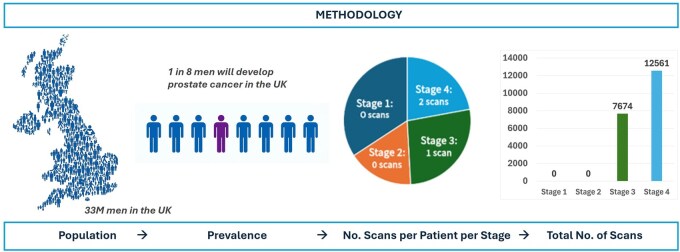
Illustration of the methodology for prostate cancer (infographic on the left hand-side adapted from Prostate Cancer UK website). Take prostate cancer incidence rates per stage and using population calculate number of people per stage; establish number of scans per stage based on clinical guidelines; multiply people/stage by scans/stage and add up to get total number of scans required for prostate cancer.

To accurately estimate the number of PET-CT scans required for each clinical indication, the number of scans necessary at different stages and substages of diagnosis was first determined according to the RCR 2022 guidance.^5^ This involves understanding how PET-CT scans are utilized at various points during the diagnostic process. For example, as illustrated in Figure 1 for prostate cancer, certain stages of cancer or disease progression may require more frequent or specialized imaging, while early-stage conditions might only necessitate fewer or more routine scans. Once the number of PET-CT scans needed for each stage and substage of diagnosis was established, these figures were then applied to the incidence rates of each condition within the population for the years 2019 and 2021. These years were chosen as the data for them is complete and also as it marks pre- and post-Covid-19 pandemic.

For the oncological indications, cancer incidence data was obtained from NHS England Cancer Statistics^19^ and Cancer Research UK^20^ websites where available, or from relevant literature when not. A breakdown of cancer incidence by stage was sourced from the Stage at diagnosis tool collected and compiled by the National Disease Registration Service (NDRS).^21^ For neurology indications, disease incidence was obtained from the NHS Digital website.^22^

By combining the stage-specific scan requirements with the known incidence rates, the total number of PET-CT scans required for each clinical indication was calculated. This approach ensures that the estimate reflects the real-world demand for PET-CT scans, accounting for the variation in scan frequency based on disease progression and the relative prevalence of each clinical condition. The result is a detailed projection of PET-CT scan needs across different clinical indications.

### Comparison of modeled estimates to current usage in England

To assess the current usage of PET radionuclides in the England, we use the Diagnostic Imaging Dataset (DID), in particular an annotated version of the Diagnostic Imaging Dataset (DID),^2^ obtained through a Freedom of Information (FOI) request to NHS England, where the data could be filtered by year, modality, radionuclide, radiopharmaceutical, and SNOMED-CT code and descriptor.

There were cases where the descriptor was unclear, in which clinical knowledge was required to determine the most appropriate indication for allocating the scans. A significant example is whole-body F-18 FDG (fluorodeoxyglucose) PET-CT, a high-volume scan used across numerous indications. Misallocation of these scans could lead to a substantial distortion in the requirement by clinical indication. For this scan type, which represents approximately 80% of cases, we relied on data from Alliance Medical Ltd (AML),^23^ which provides a breakdown by indication. This reliance on a single data source represents a potential limitation, highlighting the need for more comprehensive recording of PET-CT scans by indication in the UK. Improved data collection would allow future analyses to better understand scan utilization and ensure resources are prioritized appropriately.

### Comparison of modeled estimates to usage in other countries

To validate our predicted requirement for additional PET-CT capacity in the UK, we used a comparative approach, leveraging Finland’s PET-CT imaging data^24^ published by the Radiation and Nuclear Safety Authority (STUK) as a benchmark. Finland was selected for this validation process because of its strong healthcare system, and detailed, well-maintained data on PET-CT usage, broken down by clinical indication, making it a reliable reference for forecasting similar imaging requirements in other countries. By calculating the number of PET-CT scans per million population and scaling these figures based on disease incidence, we were able to produce an independent validation of the predicted PET-CT demand for the UK.

We first calculated the number of PET-CT scans performed per million population in Finland. This was done by analyzing data on the total number of PET-CT scans conducted for the relevant clinical indications in Finland for 2021.^24^ By dividing the total number of scans by Finland’s population size, we determined the number of PET-CT scans per million population for each clinical indication. This figure reflects the baseline utilization of PET-CT imaging in Finland’s healthcare system, offering insights into the typical demand for this imaging modality across different diseases. Finland’s well-developed PET-CT infrastructure and advanced clinical practices make this figure a reliable benchmark for healthcare systems aiming to estimate appropriate scan capacities.

While the number of scans per million population in Finland offers a general sense of PET-CT demand, it was necessary to adjust these figures for the UK context by considering differences in disease incidence between the two countries. Since the occurrence of diseases such as lung cancer, breast cancer, and NETs may vary between Finland and the UK, scaling by incidence was essential to accurately predict the UK’s specific PET-CT demand. For example, if the incidence of prostate cancer is higher in the UK than in Finland, the UK would likely require more PET-CT scans for this particular indication. The scaling process involved comparing the incidence rates in Finland^25^ and the UK.^20^ This allowed us to adjust the Finnish data on PET-CT usage per million population to reflect the relative burden of disease in the UK.

### Future demand estimates

Evidence-based estimates of current demand were incorporated into our existing model, which predicts future demand by accounting for population growth, aging demographics, the anticipated increase in PET-CT scans for dementia once disease-modifying therapies are approved, and projected changes in disease prevalence as described in Table 1.

**Table 1:** Future demand prediction assumptions.

Disease type	Drivers for future demand	References
**Prostate cancer**	Population growth; age-period-cohort model projected changes in disease prevalence	^26–28^
**Lymphoma**	Population growth; age-period-cohort model projected changes in disease prevalence	^26^ ^,^ ^27^ ^,^ ^29^
**NETS**	Assumed 5% annual growth, reflecting a recent slowdown from rapid increases over recent decades	^30^
**Lung**	Population growth; age-period-cohort projected changes in disease prevalence; stage proportions expected to change due to national screening (based on Italian long term follow up trial data).	^26^ ^,^ ^27^ ^,^ ^31^ ^,^ ^32^
**Breast**	Population growth; age-period-cohort model projected changes in disease prevalence	^26^ ^,^ ^27^ ^,^ ^33^
**Gynecological**	Population growth; age-period-cohort model projected changes in disease prevalence; impact of HPV vaccine.	^26^ ^,^ ^27^
**Dementia**	Population growth; anticipated increase once disease-modifying therapies are approved; incidence trends from 2002 to 2019 study projected to 2040.	^26^ ^,^ ^34^
**Cardiovascular**	Population growth only as future rates uncertain due to aging population and evolving medication use.	^26^

### Limitations

The modeling presented has several important limitations. First, our analysis used national-level PET-CT scan rates and did not account for regional variation. In practice, PET-CT utilization varies between regions and by clinical indication; for example, some areas perform relatively high volumes of scans for melanoma and sarcoma, while others perform very few. Local differences in clinical practice, commissioning decisions, workforce, and scanner availability further contribute to variability in actual scan volumes. Consequently, our model estimates potential demand at a national level, but regional disparities and local practice patterns may significantly influence achievable activity.

Second, comparison with Finland was used to provide a benchmark for mature adoption of cardiovascular PET-CT; however, service configurations differ substantially, and high-volume Finnish centers often perform integrated “one-stop shop” imaging protocols that may not be replicable in the UK. Differences in workforce, organizational structures, and coding practices may also influence reported scan volumes. Furthermore, the modeling assumes that all clinically indicated scans would be performed, representing an upper-bound estimate of potential demand rather than achievable capacity in the short term.

Finally, the methodology was developed and reviewed by clinical experts in each relevant domain (cardiology, oncology, neurology, etc.), who were consulted to determine the most appropriate clinical indications for PET-CT in their specialty and to verify assumptions regarding disease incidence, patient pathways, and typical scan use. Clinical input was used systematically for the primary indications included in the modeling (prostate cancer, lymphoma, NETs, lung cancer, breast cancer, gynecological cancers, dementia, and cardiovascular disease). For the remaining indications, which account for approximately 39% of PET-CT scans, clinical expertise was not applied individually due to the diversity of conditions, smaller case volumes, and limited robust guidance. This approach ensured that the model accurately captured demand for the main indications while acknowledging that the “other” indications were not modeled in detail.

These limitations should be considered when interpreting the results, as they may partially explain the gap between modeled demand and current PET-CT activity in England.

## Results and discussion

### Actual vs model predicted number of PET-CT scans in England

Table 2 shows the actual and predicted number of PET-CT scans per year for 2019 and 2021 for England; the rates of PET-CT scans for 2019 and 2021 for England per 100 000 population; and the ratio of model predictions to actuals for the year 2021; all split by clinical indication. Comparing the actuals from 2019 and 2021, an increase in the number of PET-CT scans can be observed over all the conditions included. There is also an increase in the rates of PET-CT scans per 100 000 population between 2019 and 2021, meaning that the number of scans is increasing at a higher rate than population levels. The model predictions do not all follow the same pattern, as for some conditions, the estimated number and rate of scans per 100 000 population in 2021 are lower than in 2019 (e.g., prostate cancer and lymphoma). This is because the incidence rates for prostate and lymphoma cancer decreased in 2021 compared to pre-pandemic levels,^19^ and the model takes incidence rates into account. The ratios of actuals to predicted for 2021 show that based on the model predictions there should be an increase in the number of PET-CT scans performed for dementia, prostate cancer, breast cancer, NETs, and cardiovascular conditions. The model predictions are in line with actuals for lymphoma, lung, and gynecological cancers.

**Table 2:** Actual and model predicted number of PET-CT scans per year for 2019 and 2021 for England, split by clinical indication.

	Number of PET-CT scans per year				Rates of PET-CT scans per 100K POP				Ratios
	Actuals 2019^2^	Actuals 2021^2^	Model 2019	Model 2021	Actuals 2019	Actuals 2021	Model 2019	Model 2021	Ratio actuals to predicted (2021)
**Prostate**	1575	4665*	22 357	20 235	2.80	8.25	39.76	35.78	4.34
**Lymphoma**	33 582	40 274	41 491	40 436	59.72	71.21	73.79	71.50	1.00
**Nets**	8481	11 818	26 948	27 104	15.08	20.90	47.92	47.93	2.29
**Lung**	57 356	68 787	61 356	68 584	102.00	121.63	109.12	121.27	1.00
**Breast**	3804	4562	15 459	18 540	6.77	8.07	27.49	32.78	4.06
**Gynecological**	5541	6645	6209	7447	9.85	11.75	11.04	13.17	1.12
**Dementia**	3175	3685	26 503	27 213	5.65	6.52	47.13	48.12	7.38
**Cardiovascular**	4216	5671	8432	11 342	7.50	10.03	15.00	20.05	2.00
**Rest**	–	93 259	–	140 999	–	164.90	–	249.31	1.51
					Additional number of scans				
**Total England**		23 9367		36 1900	122 533				
**Total (UK)**		26 9851		43 1109	161 258				

Rates of PET-CT scans for 2019 and 2021 for England per 100 000 population. The last column shows the ratio of model predictions to actuals for the year 2021. The last two rows show the additional number of scans required when comparing model to actuals for England and scaled up to the UK for 2021.

(*) The number of reported prostate cancer scans in 2021 in NHS England^2^ was 1665. However, in this dataset no F-18 PSMA scans appear to have been recorded (whereas F-18 choline and Ga-68 PSMA scans were recorded). Anecdotal evidence suggests that F-18 PSMA scans were carried out in England in 2021; an estimated 3000 scans have been added to the reported number of prostate scans to account for this.

**Figure 2:**
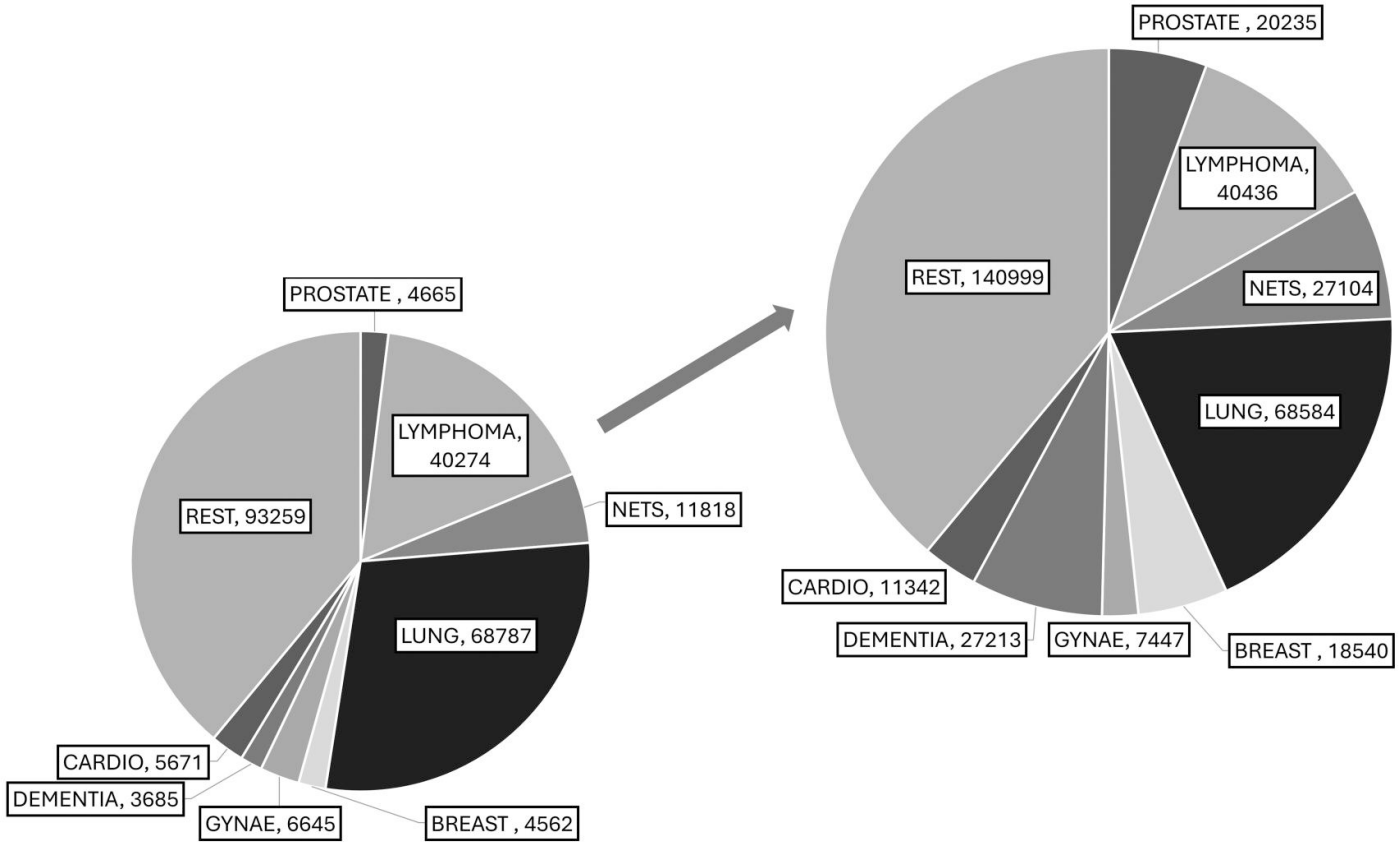
Illustration of current usage (lower left) vs. estimated demand (upper right) split by clinical condition. The pie chart areas are scaled by volume of imaging. Data for 2021.

The total number of PET-CT scans for England actuals and predicted for 2021 shown in the penultimate row of Table 2 has been calculated by adding the number of PET-CT scans recorded for each of the clinical indications under consideration plus the rest (and for ‘Actuals’ this adds up to the reported number in the DID for 2021^2^) The total number of actual PET-CT scans for the UK has been calculated using the actuals data from England plus that from the devolved nations, whereas the predicted value has been scaled by population from England’s predicted value. The additional number of scans has been calculated by subtracting predicted minus actuals. Figure 2 illustrates the current usage vs. estimated demand for the different clinical indications modeled for 2021.

### Comparison to Finland

**Figure 3:**
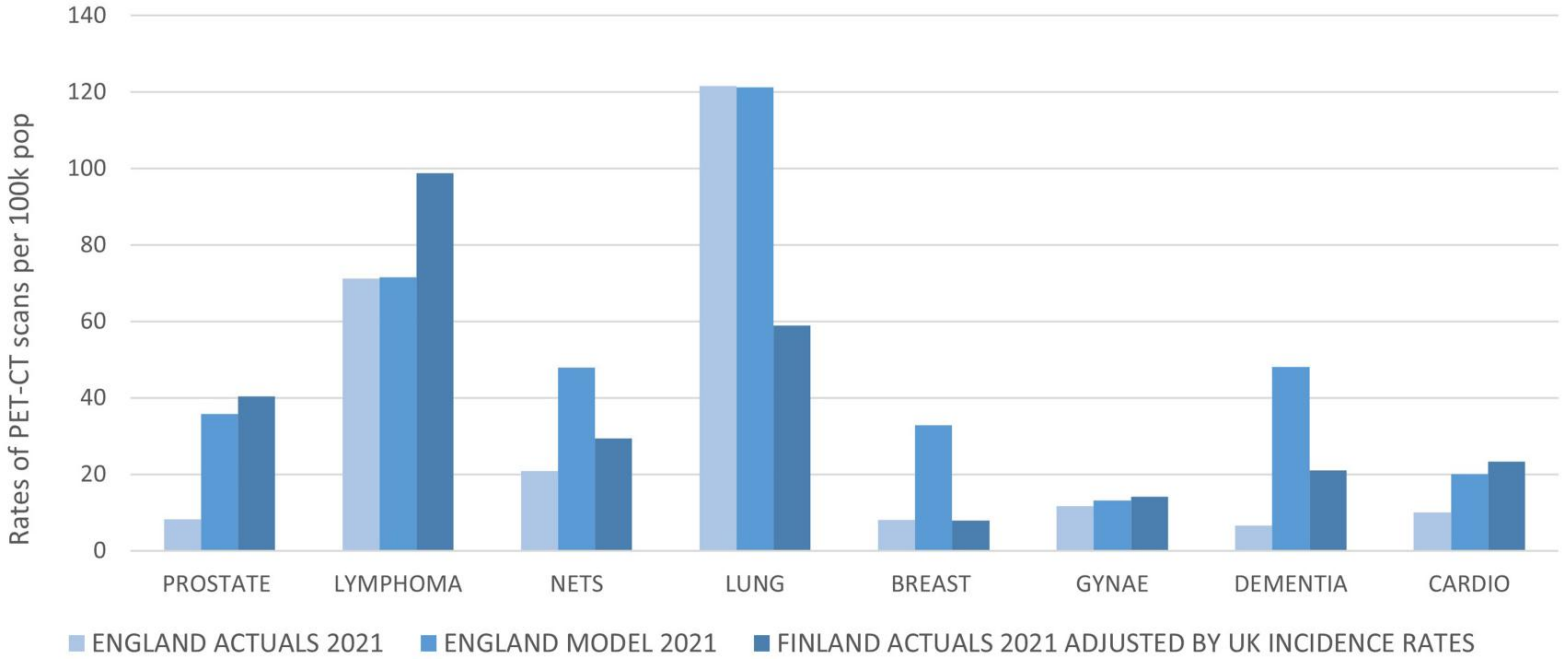
Rates of PET-CT scans per 100 000 population for England actuals and model predictions and Finland actuals adjusted by UK incidence rates for 2021.

Table 3 and Figure 3 illustrate the PET-CT scan rates per 100 000 population for 2021, comparing actual data from England, model-predicted rates for England, and actual data from Finland adjusted by UK disease incidence rates, categorized by disease type; the incidence data and adjustment calculations are summarized in Table S1. For all clinical indications except for lung and breast cancer, Finland shows higher scan rates than England, with particularly notable differences in prostate cancer, cardiovascular disease, and dementia, where Finland’s rates are significantly higher.

**Table 3:** Actual PET-CT rates per 100 000 population for England (actuals and model predicted) and Finland (actuals) adjusted by UK disease incidence rates for 2021, split by clinical indication.

	Rates of PET-CT scans per 100 000 population		
	England actuals 2021	England model 2021	Finland actuals 2021 adjusted by UK incidence rates
**Prostate**	8.25	35.78	40.35
**Lymphoma**	71.21	71.50	98.83
**Nets**	20.90	47.93	29.37
**Lung**	121.63	121.27	58.91
**Breast**	8.07	32.78	7.98
**Gynecological**	11.75	13.17	14.10
**Dementia**	6.52	48.12	21.04
**Cardiovascular**	10.03	20.05	23.36
**Rest**	164.90	249.31	28.03

For prostate and gynecological cancers, as well as cardiovascular conditions, the model-predicted rates for England align closely with Finland’s rates, suggesting that the model effectively captures the anticipated demand in these areas. In contrast, for lymphoma and lung cancer, England’s model predictions align more closely with its actual figures than with Finland’s, indicating that England’s demand for PET-CT capacity remains lower for lymphoma but higher for lung cancer compared to Finland.

For NETs, breast cancer, and dementia, the model-predicted rates for England are higher than both the actual rates for England and Finland, though for NETs and dementia, England’s model projections are closer to Finland’s higher rates. This could suggest an underutilization of PET-CT in these areas, with the model reflecting potential future increases in demand based on evolving clinical practices and guidelines.

### Future predictions

**Figure 4:**
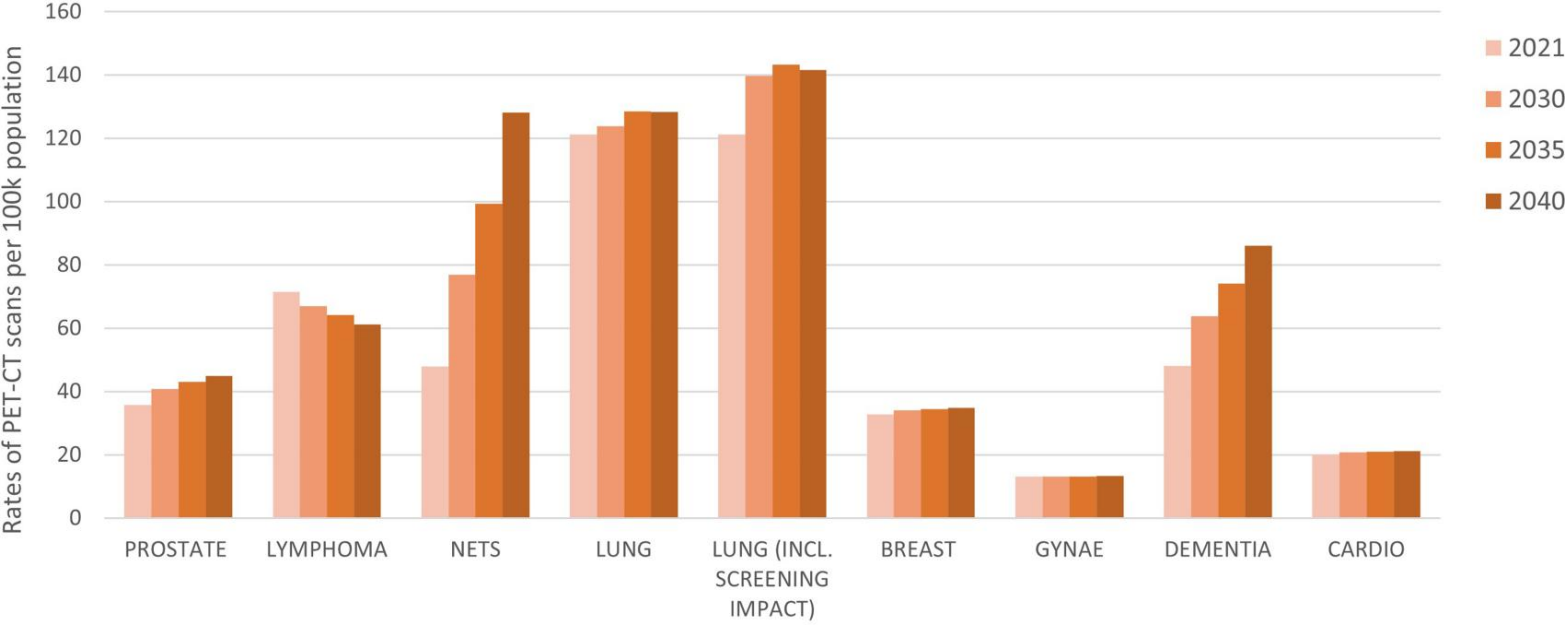
Rates of PET-CT scans per 100 000 population for England model predictions for 2021, and future predictions to 2030, 2035, and 2040.

Table 4 and Figure 4 show the model estimated PET-CT rates per 100 000 population for England for 2021, and the projections for 2030, 2035 and 2040, split by clinical indication. A sharp rise in rates is observed for NETs and dementia, driven by both the predicted increase in incidence and recent clinical guidelines recommending PET-CT for diagnosis. For prostate and lung cancers, the increase in rate is more moderate but still outpaces population growth. It is worth noting the sharper increase in the rate for lung cancer assuming the implementation of a screening program has impacted on split by stage at diagnosis. Cardiovascular and breast and gynecological cancer rates are predicted to maintain a constant rate in future predictions.

**Table 4:** Estimated PET-CT rates per 100 000 population for England for 2021, 2030, 2035, and 2040, split by clinical indication.

	Estimated PET-CT rates for England per 100 000 population			
	2021	2030	2035	2040
**Prostate**	35.78	40.75	43.03	44.88
**Lymphoma**	71.50	66.91	64.15	61.20
**Nets**	47.93	76.90	99.33	128.05
**Lung**	121.27	123.87	128.53	128.23
**Lung (with impact of screening)**	121.27	139.77	143.31	141.54
**Breast**	32.78	34.10	34.51	34.86
**Gynecological**	13.17	13.05	13.20	13.37
**Dementia**	48.12	63.82	74.14	85.98
**Cardiovascular**	20.05	20.74	20.99	21.21
**Rest**	249.31	257.89	260.97	263.62

## Conclusion

This study presents a robust model for estimating current and future PET-CT demand in England, highlighting the growing need for imaging across multiple clinical indications. The disparity between current PET-CT scan volumes in England and the modeled estimates is partly attributable to fundamental resource and funding limitations. For example, the availability of Ga-68 generators restricts the provision of PET-CT for NETs, and limited funding for amyloid PET tracers constrains dementia imaging. Other factors, such as workforce capacity, scanner availability, and organizational barriers, also contribute to under-provision. Our model represents potential clinically indicated demand rather than achievable activity within the current UK service configuration and is not intended *prima facie* as a commissioning tool for implementing current clinical care, but rather, by incorporating updated guidelines, population growth, and evolving disease prevalence, the model offers a data-driven approach to healthcare planning. The findings suggest that PET-CT demand will continue to rise, particularly for conditions such as NETs, dementia, and prostate cancer, which aligns with the findings published previously.^6–8^^,^^35^

A key insight is the importance of comprehensive and standardized data collection as highlighted also in Lynch et al.^11^ The comparison with Finland^24^ demonstrates how well-maintained national datasets can improve demand modeling and healthcare planning. Implementing a similar approach in the UK would enable more accurate national-level projections. Expanding data collection efforts beyond England would allow for UK-wide modeling, improving equity in service provision and ensuring healthcare resources are distributed according to actual patient needs.

The study also highlights the need for continuous validation of demand projections. As guidelines evolve and new therapies emerge, ongoing monitoring of PET-CT utilization is essential for refining capacity planning. Future research should assess the impact of emerging diagnostic technologies and alternative imaging modalities to ensure adaptable, evidence-based healthcare strategies.

## Supplementary Material

tzag006_Supplementary_Data
