# Supplementary material for: Assessing current and future demand for PET-CT imaging in England: a comparative analysis of 2013 and 2022 Royal College of Radiologists Guidelines
Source: BJR Open. 2026 Mar 4;8(1):tzag006. doi: 10.1093/bjro/tzag006 (PMC13020349; doi:10.1093/bjro/tzag006)
Supplement: tzag006_Supplementary_Data [file tzag006_supplementary_data.docx]

**Supplementary Material**

Table S1: Adjustment of Finnish PET-CT scan volumes to estimate UK demand based on relative disease incidence.

|  | **INCIDENCE RATES PER 100,000 POPULATION** | | **RATES OF PET-CT SCANS PER 100,000 POPULATION** | |
| --- | --- | --- | --- | --- |
|  | **ENGLAND (2019)** | **FINLAND (2021)** | **FINLAND (2021)** | **FINLAND (ADJUSTED)** |
| **PROSTATE** | 85.24 | 94.53 | 36.38 | 40.35 |
| **LYMPHOMA** | 24.33 | 31.02 | 77.54 | 98.83 |
| **NETS** | 8.66 | 8.92 | 28.50 | 29.37 |
| **LUNG** | 71.65 | 53.96 | 78.24 | 58.91 |
| **BREAST** | 86.13 | 92.68 | 7.41 | 7.98 |
| **GYNAECOLOGICAL** | 11.81 | 11.78 | 14.13 | 8.07 |
| **DEMENTIA** | 196.07 | 261.92 | 15.75 | 8.07 |
